# Among People and Their Doings

**Published:** 1895-11

**Authors:** 


### AMONG PEOPLE AND THEIR DOINGS.

The use of finely cut or ground tobacco, made into a bolus the size of an egg and placed far back in the pharynx, has been suggested as a ready and effective remedy to reduce choke in cattle, causing relaxation of the parts, with retching and violent expulsive movements.

Special milk-inspector Vandegrift has been delegated to work in the South Jersey district, where anthrax has been prevailing for a time, to guard against the sale of milk from this district until the disease is under subjection.

Seven thousand horses were recently purchased by the Portland (Oregon) Horse-meat Canning Factory at three dollars per head on the ranch. As these horses were of little use save for saddle purposes, and greatly aided in demoralizing the horse industry by being thrown upon the market in great numbers from time to time, it will prove a great aid to a better future for our breeders.

With the abandonment of the omnibus line on Broad Street, Philadelphia, will disappear the last of the horses used for street-car traction. The trolley has proved a more rapid factor in the abandonment of horses than steam did many years ago in sounding the death-knell of the old stage-coach lines.

The Board of Health of Kansas City, Mo., has established a plan of inspection of dairies supplying milk to the city. H. B. Adair, veterinarian, has been selected by the Board to decide all questions of a veterinary sanitary nature that may arise.

The value of prophylactic measures in dealing with tuberculosis finds abundant proof in Prussia, where, between the years 1887 and 1893, deaths from this disease were 70,000 less than expected from the average of the previous years.

The National Veterinary Association of Great Britain has had an experience similar to other national organizations, that there are periods when its membership decreases. This organization, some thirteen years in existence, has now a membership of but three hundred, which the *Veterinary Record* says has been exceeded in other years. At its annual meeting at Birmingham several important papers were presented.

The State Experiment Station of Missouri still continues to have no veterinarian attached to the staff. Have the live-stock interests of Missouri become of so little importance that they need no further care or assistance, or have they so far rid themselves of all serious problems of disease among their stock, of the value of economic feeding, and many other questions which are more easily solved through the assistance of a qualified veterinarian?

THE aluminum shoe, with inlaid steel plates, patented by Mr. Jerome, of Chicago, has been given a test by army officers in Arizona. Shoes which had been worn over a month in a partially rocky country were in fair condition for resetting. The price of these shoes is too high to warrant them being adopted for the service, as economy and not fitness and durability seems to be the watchword with those who purchase such supplies for the mounted service.

The shoes used in the army are the Berdan shoe, the Perkins, and the "Goodenough." The latter is considered a very serviceable shoe in a sandy country and has the advantage of being worked without heating; but has the disadvantage in the army of not having suitable nails issued for its use. The "Perkins" shoe, a light steel shoe, is used with success on the prairies. The "Berdan," an iron shoe, is issued for use in the East and in the posts situated in rocky districts.

DURING the recent war between China and Japan the latter did very effective work with their cavalry, and, as a consequence, the cavalry arm will probably be increased to a large extent. The Japanese cavalryman averages 130 pounds and uses a pony not exceeding 14.1 and not weighing over 800 pounds, as a rule. Owing to the cheapness of Oregon and Western horses, the Japanese cavalry authorities are seriously considering the idea of importing them to replace their ponies. The "Walers" and Australian horses used by the British in India are quite expensive, and were it not for John Bull's clannishness our Oregon and Montana horses could be shipped to India at half the price of Australian horses. It behooves our horse-ranchmen in the Northwest to watch these two markets, as it would be far more profitable to ship horses to Japan and India for war purposes than to ship them to China in the canned form for domestic purposes.

INFORMATION reaches us from the Orient that during the passage of the large transport ships from Japan to China, just before the battle of Wai-hai-wai (pronounced Wā-hī-wā), in the recent war, that over 600 horses out of 2500 were killed in a severe storm, owing to the fact that the Japanese tied their horses head-to-head along a rope running along the centre of the deck, thus preventing the horses from getting anything to brace themselves against, the result being that so many horses had their necks and legs broken by being thrown.

The Board of Trustees of the Clemson Agricultural College of South Carolina are said to have appointed a veterinarian on the staff at a salary of $800 per year. Surely this is not a munificent salary for a veterinarian skilled to fill such a position properly.

An outbreak of Texas fever recently occurred in a dairy herd in North St. Louis, ten deaths occurring in the same the first few days, with the balance of the herd, save one, sick. These animals were allowed to graze along the railroad tracks near the Union Stock Yards. State Veterinarian Turner, in conjunction with Dr. Starckloff, of the St. Louis Board of Health, investigated the outbreak, and the latter board has sought the official assistance of one of St. Louis' veterinarians.

A very complete matriculation-blank has been adopted by the Kansas City Veterinary College. It makes a good record-keeper, and the student signing and accepting its conditions is made aware of what is expected of him.

Anthrax among the horses, cattle, and swine is causing great losses to farmers in Missouri. Texas fever has also broken out in one or two localities along railroad lines.

State Veterinarian Turner, in his report for Missouri, refers to a loss in the entire State of 31 per cent. of the hogs from hog-cholera. He deals with the subjects of strangles, corn-stalk disease, nasal polypus, hog-cholera (the many hog-cholera nostrums come in for just and severe criticisms), locomotor ataxia, naso-fontal abscesses, and barbed-wire injuries, with remarks on quarantine regulations and a quarantine-line for splenic or Texas fever.

Dr. B. M. Freed, Sharon, Pa., a graduate of the Ontario Veterinary College, Class of 1891, has had the misfortune to have his diploma stolen from his office. Its use or purchase should be carefully guarded against by all members of the profession.

For the docking of four horses, the owner and the two persons performing the operation were recently heavily fined before a London (England) magistrate.

If castration in Great Britain reached the point of one dollar (five shillings) and rarely exceeded five dollars (one pound) as a fee, as it does in America, the performance of the operation standing would meet with little criticism, aside from the fact that often twenty-five or fifty are operated upon in a few hours, are under no excitement after the operation, are not overheated, are not cramped in their muscles from the casting and securing methods in vogue, and will walk right off in the pasture-field and go to grazing. The excision of the cord, standing or cast, is necessarily painful, but with the écraseur it is but momentary in character, and all the strength of the animal is reserved by the standing method to react from the operation.

An unusual amount of work is being done in parts of California among cattle with vaccination looking to the control and lessening of the serious losses resulting in the past from outbreaks of anthrax in certain well-defined localities.

A self-sterilizing thermometer-case is one of the latest devices in human practice to avoid any transmission of disease when used orally.

A certain well-circumscribed district in Philadelphia has been the centre for an outbreak of rabies for several successive years.

Rabies reported as prevailing to an unusual extent among the dogs and other animals by Dr. J. C. McNeil, of Pittsburg, seems to have thoroughly abated.

A local outbreak of anthrax, or Texas fever, is reported from Huntington County, Pennsylvania.

One of New Jersey's State milk-inspectors has been visiting the neighborhood of Flemington, N. J., and the arrest of sellers of impure milk followed, upon whom very severe fines were imposed. Let the good work go on.

Denmark has one of the most rigid systems of inspection of meat-products in the world.

The New York State Veterinary Medical Society has changed its application-blank in one of its chief features; it now reads: "I am registered in the County Clerk's office of . . . County on the . . . day of . . . 18 . . ." This formerly read: "I am a graduate of . . . college, year of . . . , or have been in active practice for . . . years."

Rabies has broken out in Williamsport, Pennsylvania.

Tubercular serum is the latest curative agent brought forth at a recent medical convention at Bordeaux, France, Prof. Marigliano, an Italian, claiming the discovery of its merits.

How city boards of health can longer perform their work without an official veterinarian on their staff is beyond our understanding, when such boards stand for so much on behalf of their constituents in protecting them from the dangers that lie in the meat- and milk-supply.

The political exigencies of Pennsylvania have demanded that since June 1st this State should be without the aid and assistance of an official State Veterinarian, though the duties of the State Live-stock Sanitary Board were to go into execution on June 1st. Such are the results of the infamous spoils system in its extreme methods.

St. Louis, Mo., like many other large cities, while realizing the crying need of reform in a better control of the milk-supply, that her people may be preserved from the dangers that lurk therein, seems to have become so wrapped up in the perpetuation of the spoils system in the management of her city government that this problem must wait until the issues of national politics shall have been settled through the stockholders' pockets of the city corporation. When will citizens, taxpayers, and property-owners wipe away this miserable system of city government?

In the tenth year of the reign of Henry VIII., on September 23, 1518, John Chambre, Thomas Linacre, Ferdinand de Victoria, Medicorum Nostrorum, Nicholas Holsack, John Francis, Robert Yaxley, were granted letters-patent giving them the privilege of admitting men to practise medicine in London and seven miles around. This was the original foundation of the present Royal College of Physicians of London. The first letters-patent having apparently been inadequate for the purpose intended, in the fourteenth year of Henry VIII. a statute was passed enacting that no person save a graduate of Oxford or Cambridge should practise in England, unless he had a license from the President of the College of Physicians aforesaid and from three of the "elects," who were chosen from among the fellows.—*Gentleman's Magazine.*

